# The Role of the Microbiota Gut–Liver Axis during HCV Chronic Infection: A Schematic Overview

**DOI:** 10.3390/jcm11195936

**Published:** 2022-10-08

**Authors:** Nadia Marascio, Carmen De Caro, Angela Quirino, Maria Mazzitelli, Emilio Russo, Carlo Torti, Giovanni Matera

**Affiliations:** 1 Clinical Microbiology Unit, Department of Health Science, “Magna Graecia” University, 88100 Catanzaro, Italy; 2 System and Applied Pharmacology, Department of Health Science, “Magna Graecia” University, 88100 Catanzaro, Italy; 3 Infectious and Tropical Diseases Unit, University Hospital of Padua, 35128 Padua, Italy; 4 Infectious and Tropical Diseases Unit, Department of Medical and Surgical Sciences, “Magna Graecia” University, 88100 Catanzaro, Italy

**Keywords:** HCV, microbiota, gut–liver axis, dysbiosis

## Abstract

Hepatitis C virus (HCV) still represents one of the most important worldwide health care problems. Since 2011, direct-acting antiviral (DAA) drugs have increased the number of people who have achieved a sustained virological response (SVR). Even if the program to eradicate HCV by 2030 is still ongoing, the SARS-CoV-2 pandemic has created a delay due to the reallocation of public health resources. HCV is characterized by high genetic variability and is responsible for hepatic and extra-hepatic diseases. Depending on the HCV genotype/subtype and comorbidities of patients, tailored treatment is necessary. Recently, it has been shown that liver damage impacts gut microbiota, altering the microbial community (dysbiosis) during persistent viral replication. An increasing number of studies are trying to clarify the role of the gut–liver axis during HCV chronic infection. DAA therapy, by restoring the gut microbiota equilibrium, seems to improve liver disease progression in both naïve and treated HCV-positive patients. In this review, we aim to discuss a snapshot of selected peer-reviewed papers concerning the interplay between HCV and the gut–liver axis.

## 1. Introduction

The World Health Organization (WHO) planned, among other things, to reduce new infections and deaths related to hepatitis C virus (HCV) by 2030 [1]. Since 2011, the introduction of direct-acting antiviral (DAA) drugs in clinical practice has significantly increased the rate of people who have eradicated the virus, although a small percentage of positive patients are still difficult to treat [2]. The HCV eradication program is ongoing, even if the severe acute respiratory syndrome coronavirus 2 (SARS-CoV-2) pandemic has created a delay in the diagnosis and cure of patients with HCV infection due to a reallocation of public health measures and resources [3,4]. Van Dijk and co-authors, by applying a mathematical model, have shown an increased number of hepatic diseases during the COVID-19 pandemic scenario [4]. However, timely diagnosis and DAA treatment has reduced the pathogenic effects of a persistent HCV infection, in both hepatic and extra-hepatic diseases [5]. In 2020, the European Association for the Study of the Liver (EASL) reported the latest update of recommendations to treat HCV-positive patients [2]. The wide spectrum of symptoms due to HCV variability and patients’ clinical history requires tailored treatment, taking into account both the health status and comorbidities of the patient population [6,7]. Recently, it was demonstrated that the intestinal microbiota is associated with the outcome of hepatic diseases [8]. An increasing number of studies have analyzed the relationship between viral hepatitis and the gut microbial community [9,10]. In particular, HCV impacts the microbiota gut–liver axis by altering the composition of gut microbiota (dysbiosis), characterized by a loss of microbial diversity and the expansion of potential pathogens [11,12]. Viral eradication produces a beneficial effect on this axis, mitigating inflammation and liver stiffness by reducing dysbiosis [13]. However, the interplay between HCV infection and the gut microbiota is poorly understood and controversial, especially because external influences on the microbiota cannot sufficiently be controlled in human beings, as it can in animal models [8,14,15].

Starting with the main epidemiological and pathogenic characteristics of HCV, in this narrative review, we would like to shed light on gut microbiota taxonomy influenced by viral chronic infection and its clinical follow-up. The literature research was conducted by using the PubMed database, taking into account articles written in English, but without a specific time window in order to display a clear and schematic overview.

## 2. HCV Infection: Epidemiology and Pathogenesis

To date, HCV infection remains a major global health issue. Estimates from the WHO count more than 58 million people having a chronic HCV infection, with a further 1.5 million new infections occurring every year [1]. The same estimates report almost 300,000 deaths due to HCV complications, such as liver cirrhosis (LC) or cancer [1]. Regions with the highest burden of disease are the Mediterranean areas, Southeast Asia, Africa, and some regions of the Americas [1].

HCV is a blood-borne RNA virus, lacking a proofreading activity during its replication, which thereby increases the likelihood of viral mutations and pathogenicity [16]. There are eight main genotypes and numerous subtypes, whose prevalence is largely geographically differentiated. HCV1 is very common across Europe and the United States, whereas HCV2, HCV4, and HCV5 genotypes are very common in African countries, and HCV2 and HCV6 are mainly predominant in Asia [17]. HCV7 is responsible for less than 1% of total HCV infections, and HCV8 was identified for the first time in patients living in Canada [5].

HCV transmission occurs by four main routes: blood transfusion, sharing of unsafe needles, syringes among intravenous drug users, unprotected sexual intercourse, and vertical transmission [18]. Although transmission by the vertical route and blood products significantly decreased over the last few decades, it is increasing among those who experience unprotected sex (especially among men who have sex with men) and intravenous drug abusers [19]. The risk of transmission is further increased by the presence of other viral or bacterial sexually transmitted infections, such as human immunodeficiency virus (HIV), syphilis, and gonorrhoea [19].

The possible evolution of HCV primary infection is related mainly to viral characteristics and host factors [20]. In most cases, primary infection is asymptomatic or has a non-specific onset symptomatology, whereas only a minority of acute cases are serious, leading, in the worst-case scenario, to fulminant hepatitis [21]. In 15–25% of cases the infection clears spontaneously, whereas in 75–85% it becomes chronic, especially in subjects who have some risk factors (e.g., HIV co-infection) for the inability of the immune system to clear it [21]. This also happens for the ability of HCV to counteract the retinoic acid-inducible gene-1 (RIG-1) pathway and to evade the immune challenge [22,23]. Liver disease progression is still being debated and seems to be related to specific host risk factors, such as age or alcohol abuse [24]. Although HCV has a major liver tropism, chronic infection may result in systemic disease involving several other systems [6]. It is often associated with weight loss, fatigue, nausea, abdominal pain, neuropsychiatric symptoms (such as depression), lympho-prolypherative disorders (e.g., B cell non-Hodgkin lymphoma), renal diseases, diabetes, cardiovascular and cerebrovascular diseases, and cryoglobulinaemic vasculitis [6]. HCV pathogenesis is associated with viral variability. The HCV3 genotype, inducing lipid accumulation, is significantly associated with steatosis compared to the HCV1 genotype [25]. The HCV1b subtype is capable of establishing chronic viral infection, while both HCV1 and HCV2 genotypes enhance the risk of kidney disease [5,26].

**Figure 1:**
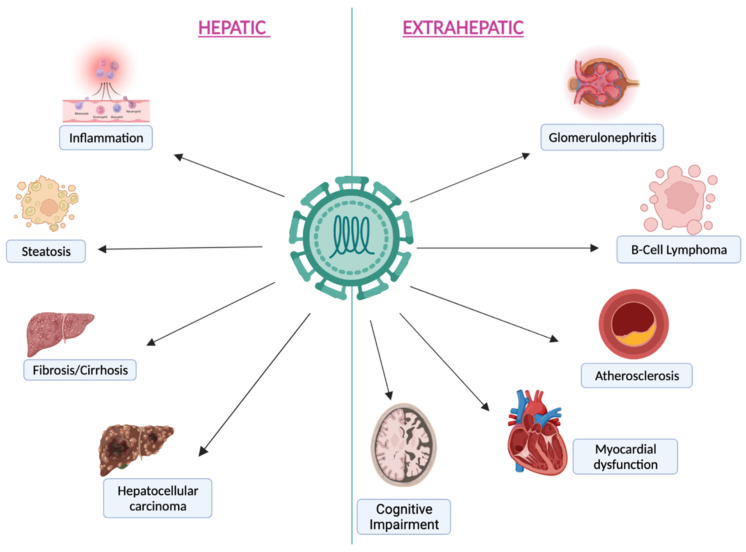
The principal hepatic and extra-hepatic manifestations during chronic HCV infection.

As previously mentioned, following chronicity, and above all if left untreated, HCV infection progressively leads to cirrhosis due to a locally driven, virus-specific T cell immune response rather than a direct viral cytopathic effect [27]. HCV is not a direct oncogenic virus [15]. The development of hepatocellular carcinoma (HCC) is mediated by viral proteins, such as NS5B, binding the retinoblastoma tumor suppressor protein, or NS2 and core proteins, influencing cycle progression. Finally, viral products influence intracellular pathways triggering cellular proliferation [15]. The risk of developing HCC, even if reduced, still persists after HCV eradication, especially in subjects with advanced liver disease [28,29]. For this reason, surveillance for HCC is usually performed in those who report a long history of chronic HCV infection and have advanced liver disease before treatment. Furthermore, the presence of co-infection with other hepatic viruses and/or HIV accelerates disease progression and liver damage due to increased local inflammation [5,20]. The principal clinical courses of HCV infection, including hepatic and extra-hepatic diseases, are summarised in Figure 1.

## 3. HCV Infection Effect on the Gut–Liver Axis

The gut–liver axis represents the link between the gut microbiota and the liver, where both communicate via the portal vein, systemic circulation, and biliary tract [30]. The portal vein provides about 70% of the blood to the liver, transporting nutrients and metabolites from the gut to the liver [31]. However, this route also transports toxic products such as: peptidoglycans, endotoxins or intact bacteria, which may disrupt the liver’s metabolic functions [32]. Furthermore, the liver is responsible for bile acid (BAs) synthesis from cholesterol via 17 liver enzymes which are secreted in the biliary tract and reach the small intestine via the duodenum, combining with other components along the biliary tract and enabling the emulsification, digestion, and absorption of dietary fats. BAs are known to be significant regulators of lipid metabolism, glucose and energy homeostasis and are also involved in the regulation and communication of the gut–liver axis [33]. Approximately 95% of bile acids are reabsorbed at the terminal ileum level and return to the liver via the hepatic portal vein (the enterohepatic circulation) [34]. The residual 5% of BAs are deconjugated, dehydroxylated, and dehydrogenated by the colonic microbiota and progress to secondary bile acids (deoxycholic acid, lithocholic acid, and ursodeoxycholic acid) that arrive at the liver and subsequently the portal circulation through passive absorption [35]. This conversion is mediated by different gut bacteria, mainly *Clostridiales* [36]. Bile acids have several roles: food digestion, integrity of the gut mucosa, and antimicrobial activity against pathogens [37]. Kakiyama and coworkers demonstrated that HCV infections (e.g., cirrhosis and advanced liver diseases), are due to the decrease in primary to secondary bile acid conversion [38]. However, it was hypothesized that this effect was related to a reduction in microbial diversity and an increase in the abundance of specific microbial taxa such as the Proteobacteria phylum, *Enterobacteriaceae* family and the genera *Staphylococcus* and *Enterococcus* [13]. Moreover, it was noted that alteration of the intestinal barrier can expose the liver to noxious compounds coming from the intestine, which may cause liver damage such as alcoholic liver disease, primary biliary cholangitis and LC. Thus an increase in gut permeability and the alteration of gut microbiota may promote further liver damage [39]. These alterations may induce an inflammatory condition and metabolic disturbance that have different effects on gut and liver health and contribute to the progression of disease, with a large release of pathogen-associated molecular patterns (PAMP) that have different effects on immune cells and hepatocytes [39].

HCV alters the pathophysiology of the liver and decreases bile production, which is reflected in pro-inflammatory bacterial overgrowth and in the microbial community [40]. BA dysregulation plays an important role in the progression of cirrhosis to liver cancer [41]. HCV infection induces an unfavorable gut microenvironment and the reduction of *Ruminococcaceae* and *Lachnospiraceae*, which produce fecal short-chain fatty acids (SCFAs) crucial to maintaining metabolic homeostasis, integrity of the intestinal barrier and differentiation of Treg cells [15,42]. On the other hand, HCV infects gut B-lymphocytes decreasing IgA levels and increasing intestinal permeability, thereby allowing bacterial translocation [43]. The increase in intestinal permeability and the transition of lipopolysaccharide (LPS)-generated liver inflammatory reactions through TLR4, promotes HCC progression, especially in subjects with chronic alcohol consumption [44]. High levels of cytokines, IgA and T cells during chronic HCV infection can control the gut community diversity; in particular, the abundance of *Prevotella* appears to be related to inflammatory mediator IL-17 [45].

**Figure 2:**
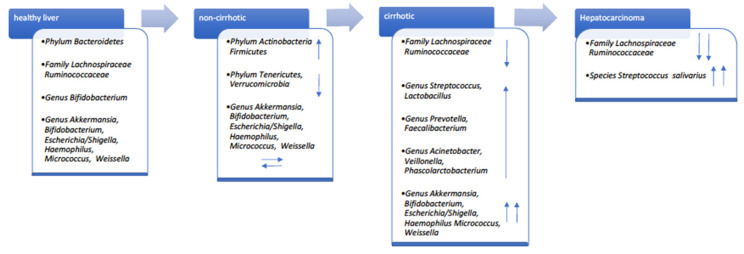
Gut taxonomy increased and decreased prevalence (blue arrows) according to liver disease stage of patients versus healthy controls.

Few studies have evaluated the gut microbiota composition of subjects with HCV infection versus healthy individuals [42,45,46]. In 2016, Aly and colleagues described for the first time a different proportion of *Firmicutes*/*Bacteroidetes* phyla, biased towards *Bacteroidetes,* in the colon environment. Infected and healthy individuals shared 22 distinct operational taxonomic units (OTUs) [45]. It is noteworthy that some specific genera, such as *Prevotella*, *Acinetobacter*, and *Veillonella* were more abundant in clinical stage 4 HCV patients, whereas *Ruminococcus* and *Clostridium* were more represented in healthy controls. Alpha diversity was lower in HCV patients [45]. In agreement with this study, Shannon entropy showed low diversity in non-cirrhotic HCV-positive patients compared to healthy controls, and in cirrhotic HCV-positive compared to non-cirrhotic patients when analyzing results from 145 individuals [47]. Interestingly, microbial composition was principally influenced by persistent HCV1 infection. Specific microbial genera, *Veillonella*, *Haemophilus*, *Streptococcus,* and *Lactobacillus* were associated with LC. Severity/stage of liver disease and HCV infection were directly related to microbial diversity [47]. HCV status and CH or LC or HCC were compared by Inoue and colleagues [42]. A significant increase in *Streptococcus salivarius* and reduction of the *Ruminococcaceae* and *Lachnospiraceae* families was found in cirrhotic patients [42]. It is likely that *S. salivarius* accelerates HCC progression by downregulating the pro-inflammatory response [48]. In Figure 2, we report the different prevalence of the main taxa according to liver diseases in patients compared to healthy subjects.

## 4. Gut Microbiota and HCV Therapy

Until 2011, PEGylated-interferon (PEG-IFN) plus ribavirin (RBV) led to sustained virological response (SVR) with a success rate of about 54–56% in HCV patients. HCV genotype/subtype was a predictive parameter for SVR [49]. In the last nine years, anti-HCV therapy has improved due to the availability of direct-acting antiviral (DAA) drugs, which replaced in clinical practice the standard of care (SOC) treatment [50]. The NS3/4A, NS5A, and NS5B polymerase nonstructural proteins (NSs) are the direct targets of therapy [7]. NSs are important for viral replication, NS3 protease, and its cofactor NS4A, which catalyzes cleavage of viral polyprotein. NS5B is the RNA-dependent RNA polymerase (RdRp). NS5A contributes to viral replication and interacts with IFN-alpha protein kinase [51]. Approximately 5% of DAA treated patients do not achieve SVR due to resistance-associated substitutions (RASs) specific for each genotype/subtype. RASs can be selected by drug pressure on genomic target regions [52].

Since 2015, several DAA IFN-free regimens have been approved [50]. In 2017, the US Food and Drug Administration (FDA) approved a therapy effective against all HCV genotypes (pan-genotypic treatment) [53]. Effective and safe DAA combinations can eradicate the virus in both previously treated and treatment-naïve patients, with more than 95% subjects achieving SVR [7]. The HCV eradication improves stages of fibrosis and liver stiffness within weeks or months. The impact of therapy on the gut–liver axis seems to be directly dependent on the stage of fibrosis and on the evaluation time after treatment [8,13].

Bajaj and colleagues reported a significant gut dysbiosis when comparing healthy controls and HCV cirrhotic patients [54]. Notably, no significant differences were found among HCV patients between groups with or without SVR. Despite the low number of patients achieving SVR with SOC therapy in 15 months, the authors suggested an improvement of microbial gut equilibrium after HCV eradication [54]. In 2021, Wellhöner and coauthors demonstrated a strong association between chronic hepatitis C and the intestinal microbial community [8]. Patients achieving SVR improved bacterial diversity and microbial community structure compared to their baseline clinical status. In particular, an improvement in bacterial diversity was observed in SVR patients without cirrhosis, whereas in SVR patients with cirrhosis no changes were found before or after DAA treatment [8]. Patients without LC showed significant changes within 24 weeks after the end of treatment. In patients without cirrhosis, the relative abundances of *Collinsella* and *Bifidobacter* genera between baseline and SVR24/48 increased significantly. At SVR24/48 in patients with LC, *Acidaminococcus* spp., *Eubacterium* spp., and *Lachnospiracea* spp. were abundant. By contrast, *Citrobacter* spp., *Enterobacter* spp., *Enterococcus* spp., *Megasphaera* spp., and *Pseudomonas* spp. were less abundant. The remodeling of the gut microbial community structure is dependent on the fibrosis stage of patients resolving chronic infection [8]. However, it cannot be excluded that the restructuring of gut microbiota requires more time in patients with cirrhosis. As reported in a small cohort study, SVR patients with cirrhosis showed improvement in the diversity and structure of gut bacterial community after one year [13]. Following DAA treatment, gut microbiota community change was independent of intestinal barrier permeability and probably related to cure of the infection and improvement in liver function, increasing BA production [55]. Viral eradication reduced the abundance of potential pathogenic bacteria, such as the *Enterobacteriaceae*, *Staphylococcus*, and the *Veillonellaceae* [13], but did not reduce signs of inflammation four years after LC regression, suggesting that bacterial translocation and intestinal inflammation may continue to exert a pro-inflammatory stimulation [56]. Even if production of inflammatory cytokines and chemokines is downregulated by viral eradication, DAA treatment does not affect the gut barrier, so the intestinal dysfunction is not totally restored [13].

In a pilot sub-study, gut microbiota of 58 HCV positive patients treated with glecaprevir/pibrentasvir (GLE/PIB) was analyzed at baseline and at 12 weeks post-treatment. Microbial diversity decreased over time, although no significant changes in gut microbiota alpha- and beta-diversity were demonstrated after DAA treatment [57].

Recently, to avoid impact of treatment, the gut microbiota in a cohort of naïve patients was analyzed at the time of diagnosis. In contrast with previous literature, HCV infection in treatment-naive patients was associated with increased diversity of microbiota and the depletion of *Bacteroidetes* phyla and *Streptococcus* genus [12]. These contrasting results are probably due to the stages of disease analyzed, antiviral treatment, HCV genotypes, and demographic characteristics of the cohort. However, HCV-associated dysbiosis could be mitigated by modulating the gut microbial community to prevent a more severe illness [12]. A summary of papers considering therapy, liver status, and gut microbial composition is reported in Table 1.

## 5. Gut Microbiota and Therapeutic Manipulation

HCV eradication achieving SVR improves inflammation and intestinal dysbiosis in the majority of treated patients. Current DAA therapy could be potentiated in order to improve control of extrahepatic and liver-associated complications, by using probiotics, prebiotics, or an appropriate diet [12,46]. Dietary food intake is the first cause of changes in the intestinal flora. Gut microbiota can rapidly change its composition under specific dietary pressure. An animal-based diet, for instance, decreases the abundance of *Firmicutes* and increases the prevalence of bile-tolerant microorganisms [58].

The effects of probiotics and prebiotics are mainly reported in animal models. Probiotics have a beneficial effect on liver disease, *Lactobacillus casei* reduces plasma levels of LPS-binding protein (LBP). *Bifidobacterium* decreases fat accumulation in the liver. In patients with LC, a combination of *Lactobacillus* spp., *Bifidobacterium* spp., and *Streptococcus* spp. is effective in preventing secondary hepatic encephalopathy [59]. The prebiotic fructo-oligosaccharides (FOSs) restore gut microbiota composition and intestinal barrier function. Lactulose increases the growth of *Bifidobacterium* and decreases LPS in serum [59]. Fecal microbiota transplantation (FMT) is able to improve gut dysbiosis and reduce hospitalization in patients with LC [60]. In addition, FMT following rifaximin antibiotic therapy could remove *S. salivarius* and increase abundance of healthy microbiome for patients in broad clinical stages. Rifaximin reduces endotoxemia, secondary bile acids, and harmful metabolite levels [61]. To cure dysbiosis by using classical approaches, next-generation phage therapy has been proposed. Clinical trials are underway to reduce pathogenic bacterial species in the gut community [62].

## 6. Conclusions

In this review, we reported the main characteristics of HCV and the effect treatment has on the gut community. Chronic HCV infection is related to a significant reduction in microbial diversity in the gut microbiota compared to healthy control groups. In this regard, microbiota composition may be used as a biomarker. DAA-tailored treatment and therapeutic manipulation of microbiota could be used in combination to improve disease progression and quality of life of infected subjects. To clearly understand and define the role of the gut–liver axis during HCV chronic infection, further investigation is needed. Despite the increasing number of papers, there are few original articles investigating the role of this axis and show some limitations. In conclusion, we propose the following suggestions for future studies.

- Study designs should be performed taking into account dietary features and geographic location of positive patients.
- The control group of non-infected subjects should be matched for age, gender, and diet.
- Experimental studies should be designed according to HCV genotype/subtype and liver disease status of positive patients.
- Pre-clinical and clinical studies, including a large cohort of patients, are required to better understand the link between the gut and liver during chronic infection.

## Figures and Tables

**Table 1:** Antiviral treatments and their effect on gut diversity of HCV infected patients.

Antiviral Therapy	Liver Status	Gut Composition and Time of Evaluation
SOC	Cirrhosis	Improvement of dysbiosis 15 months later SVR [54]
DAA	Cirrhosis	Improvement of dysbiosis 12 months later SVR [13]
DAA	Cirrhosis	No significant bacterial diversity between baseline and 24/48 weeks later SVR [8]
DAA	No cirrhosis	Improvement of dysbiosis 24/48 weeks later SVR [8]
DAA	Cirrhosis/No cirrhosis	No significant bacterial diversity between baseline and 12 weeks later SVR [57]

Legend: SOC, standard of care; DAA, direct-acting antiviral; SVR, sustained virological response.

## Data Availability

Not applicable.
